# *In Silico* Screening and Molecular Dynamics Simulation of Disease-Associated nsSNP in TYRP1 Gene and Its Structural Consequences in OCA3

**DOI:** 10.1155/2013/697051

**Published:** 2013-06-19

**Authors:** Balu Kamaraj, Rituraj Purohit

**Affiliations:** ^1^School of Bio Sciences and Technology (SBST), Bioinformatics Division, Vellore Institute of Technology University, Vellore, Tamil Nadu 632014, India; ^2^Human Genetics Foundation, Torino, Via Nizza 52, 10126 Torino, Italy

## Abstract

Oculocutaneous albinism type III (OCA3), caused by mutations of TYRP1 gene, is an autosomal recessive disorder characterized by reduced biosynthesis of melanin pigment in the hair, skin, and eyes. The TYRP1 gene encodes a protein called tyrosinase-related protein-1 (Tyrp1). Tyrp1 is involved in maintaining the stability of tyrosinase protein and modulating its catalytic activity in eumelanin synthesis. Tyrp1 is also involved in maintenance of melanosome structure and affects melanocyte proliferation and cell death. In this work we implemented computational analysis to filter the most probable mutation that might be associated with OCA3. We found R326H and R356Q as most deleterious and disease associated by using PolyPhen 2.0, SIFT, PANTHER, I-mutant 3.0, PhD-SNP, SNP&GO, Pmut, and Mutpred tools. To understand the atomic arrangement in 3D space, the native and mutant (R326H and R356Q) structures were modelled. Finally the structural analyses of native and mutant Tyrp1 proteins were investigated using molecular dynamics simulation (MDS) approach. MDS results showed more flexibility in native Tyrp1 structure. Due to mutation in Tyrp1 protein, it became more rigid and might disturb the structural conformation and catalytic function of the structure and might also play a significant role in inducing OCA3. The results obtained from this study would facilitate wet-lab researches to develop a potent drug therapies against OCA3.

## 1. Introduction

Oculocutaneous albinism type 3 (OCA3) is an autosomal recessive disorder characterized by reduced biosynthesis of melanin pigment in the hair, skin, and eyes [MIM 203290]. This disorder is mostly caused by the genetic mutation in TYRP1 gene. OCA3 is also known as Rufous oculocutaneous albinism. The human TYRP1 gene consists of 8 exons and 7 introns, spanning almost 15–18 kb of genomic DNA in the region of 9p23 [1–4]. This gene encodes a protein called Tyrosinase-related protein 1 (Tyrp1), has a molecular weight of ~75 kDa, and appears to be the most abundant melanosomal protein of the melanocyte [5, 6]. Tyrp1 is comprising of 537 amino acid residues and shares 40–52% of amino acid homology to tyrosinase. The tyrosinase-related family includes tyrosinase, tyrosinase-related protein 1 (Tyrp1), and tyrosinase-related protein 2 (Tyrp2) involved in this enzymatic process that converts tyrosine to melanin pigments. Certainly, two types of melanin are produced by melanocytes, which are pheomelanins (red or yellow) and eumelanins (brown or black) [7]. The first two steps of both eumelanin and pheomelanin production involve tyrosinase catalysing the conversion of tyrosine to 3,4-dihydroxy-L-phenylalanine (DOPA) and of DOPA to DOPA quinone [8, 9]. Then pheomelanogenesis seems to be the default pathway in the absence of MC1R signalling, with a low tyrosinase activity and a high concentration of thiolic compounds, such as cysteine. In another way, eumelanin synthesis requires *α*-MSH binding to MC1R [10, 11], which transcriptionally activates tyrosinase and upregulates Tyrp1 and Tyrp2 [12–14]. In addition to their roles in pigmentation, tyrosinase family proteins also influence the biology of melanocyte and melanoma. There is evidence that Tyrp1 is involved in the maintenance of melanosome structure and affects melanocyte proliferation and cell death [15–18].

Based on its homology to tyrosinase, Tyrp1 has also been speculated to be another tyrosinase or reveal the tyrosinase-like activity. Tyrp1 shows tyrosine hydroxylase activity, albeit under low substrate (L-tyrosine) concentration, but no DOPA oxidase activity [19, 20]. Based on that human Tyrp1 is involved in conversion of L-tyrosine to DOPA with low turnover rates, sufficient to prime the system by the generation of low amounts of DOPA, a necessary co-factor for tyrosinase activity [21]. Tyrp1 has also been attributed with various other catalytic functions including dopachrome tautomerase (Dct), dihydroxyindole (DHI) oxidase [22] and 5,6-dihydroxyindole-2-carboxylic acid (DHICA) [23]. Mutagenesis studies have recently confirmed that Tyrp1 is actively involved in inactivation of the catalytic activity of tyrosinase [24]. Observing the more number of pathological genetic variants and their structural and functional aspects of OCA3 will aid in development of personalized medicine. 

**Figure 1:**
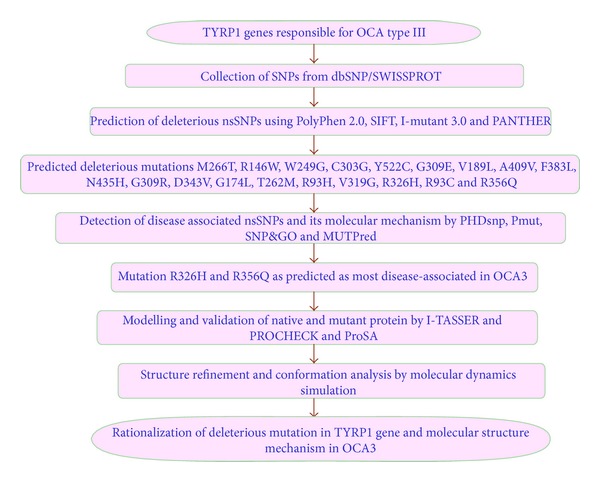
Flow chart of mutational analysis of OCA3.

Several computational algorithms used for the accurate prediction of OCA3 uncharacterized alleles for their disease related property. Mutations involved in OCA3 disorder are hard to scrutinize using *in vivo* examinations. Hence, an efficient experimental design specific to these diseases are mandatory to observe the disease associated mutation of respective SNPs. Several research articles have stated is effectiveness in identifying the deleterious and disease-associated mutations, thus predicting the pathogenic nsSNPs in correlation to their functional and structural damaging properties [25–28]. Computational studies have previously provided an efficient platform for evaluation and analysis of genetic mutations for their pathological consequences and in determining their underlying molecular mechanism [27–33]. Moreover the conformational changes in the 3D structure of the protein account for the changes in its time dependent physiological affinities and various biochemical pathway alterations [34–37]. Here we used set of computational platforms that utilizes sequence-based conservation profile, homology-based structure profile information, and support vector algorithm used to examine the disease associated nsSNPs. In this study we have applied a set of tools like PolyPhen 2.0 [38], SIFT [39], I-mutant 3.0 [40], PANTHER [41], PhD-SNP [42], SNP&GO [43], Pmut [44], and MutPred [45] to show greater accuracy for the prediction of most disease-associated mutations in OCA3 gene and their structural consequence. Further, we carried out molecular dynamic simulations (MDS) to analyse the molecular and structural basis of predicted disease associated nsSNPs. MDS were applied to observe the motion trajectory and atomic interaction of native and mutant (R326H and R356Q) Tyrp1 protein. The overall strategy implemented in this work is shown in Figure 1.

## 2. Materials and Methods

### 2.1. Dataset

The data on human TYRP1 genes were collected from OMIM [46] and Entrez gene on National Center for Biotechnology Information (NCBI) Website. The SNP information of TYRP1 gene was obtained from dbSNP (http://www.ncbi.nlm.nih.gov/snp/) [47] and Swissprot databases [48–50]. The amino acid sequence of Tyrp1 protein was retrieved from the Uniprot database (Uniprot ID: P17643). In order to build the mutant structures, we induced the point mutations in the position of 326 and 356 of Tyrp1 protein using SPDB viewer package [51]. These structures were energetically optimized by applying the all atom OPLS force field available in GROMACS package 4.5.3 [52]. 

### 2.2. Disease Related SNP Prediction

The single nucleotide polymorphism occurring in the protein coding region may lead to the deleterious consequences and might affect its 3D structure. Here we applied PolyPhen 2.0 [38], SIFT [39], I-Mutant 3.0 [40], PANTHER [41], PhD-SNP [42], SNP&GO [43], Pmut [44], and MutPred [45] tools in order to examine the disease-associated nsSNP occurring in the Tyrp1 protein coding region. PolyPhen 2.0 is based on combination of sequence and structure based attributes and uses naive Bayesian classifier for the identification of an amino acid substitution and the impact of mutation. The output levels of probably damaging and possibly damaging were classified as functionally significant (≤0.5) and the benign level being classified as tolerated (≥0.51) [38]. SIFT prediction is based on the sequence homology and the physicochemical properties of amino acids which are dictated by the substituted amino acid. SIFT score ≥0.05 indicates the amino acid substitution is intolerant or deleterious, whereas the score ≤0.05 predicted it as tolerant [39]. I-Mutant 3.0 is a support vector machine (SVM) based tool. We used the sequence based version of I-Mutant 3.0 that classifies the prediction into three classes: neutral mutation (−0.5 ≤ DDG ≥ 0.5 kcal/moL), large decrease (<−0.5 kcal/moL), and a large increase (>0.5 kcal/moL). The free energy change (DDG) predicted by I-Mutant 3.0 is based on the difference between unfolding Gibbs free energy change of mutant and native protein (kcal/moL) [40]. PANTHER program is a protein family and subfamily database which predicts the frequency of occurrence of amino acid at a particular position in evolutionary related protein sequences. The threshold subPSEC score of −3 has been assigned below which the predictions are considered as deleterious [41]. We filtered the nsSNPs that were combinedly predicted to be deleterious and damaging from these four servers. Further we used PhD-SNP, SNP&GO, Pmut, and MutPred tools to examine the disease-associated nsSNPs. PhD-SNP is SVM based classifier, trained over the million amino acid polymorphism datasets using supervised training algorithm [42]. It predicts whether the given amino acid substitution leads to disease associated or neutral along with the reliability index score [42]. SNP&GO retrieves data from protein sequence, evolutionary information, and functions as encoded in the gene ontology terms [43]. Pmut is a neural network based program which is trained on large database of neutral and pathological mutations [44]. Pmut uses 3 parameters including mutation descriptors, solvent accessibility, and residue and sequence properties to calculate the pathogenicity indexes of given input mutation data ranging from 0 to 1. The mutations with index score greater than 0.5 are predicted to be pathologically significant [44]. MutPred is a web based tool, used to predict the molecular changes associated with amino acid variants [45]. It uses SIFT, PSI-BLAST, and Pfam profiles along with some structural disorder prediction algorithms, including TMHMM, MARCOIL, I-Mutant 2.0, B-factor prediction, and DisProt [45]. Functional analysis includes the prediction of DNA-binding site, catalytic domains, calmodulin-binding targets, and posttranslational modification sites [45]. Combining the scores of all four servers, the accuracy of prediction rises to a greater extent and finally we filtered the most disease-associated mutation.

### 2.3. Modelling of Native and Mutant TYRP1 Proteins

According to the annotated information available in UNIPROT entry-P17643, the predicted deleterious mutation sites of Tyrp1 protein were observed in the topological domain which comprised between the regions 190–385. Hence, we modeled Tyrp1 protein segment which consists of 196 amino acid residues by I-TASSER server [53]. This program works by combining the folds and secondary structure by profile-profile alignment threading techniques for non-aligned regions. For the submitted sequences, five 3D models were obtained and the best model was selected based on the lowest energy. Further the native structure was mutated with the most deleterious substitution predicted in this study. In order to build the mutant structures, we made a point mutation in native Tyrp1 protein at R326H (arginine to histidine) and R356Q (arginine to glutamine) using SPDB viewer [51]. The native and mutant structures were energetically optimized by applying the all atom OPLS force field available under the GROMACS 4.5.3 package [52]. The quality of model structures was verified using the PROCHECK [54] and PROSA [55] programs.

### 2.4. Molecular Dynamics Simulation

Molecular dynamics simulation was performed by using gromacs 4.5.3 package [52] running on a single Intel Core2Duo machine with 3 GB RAM and running Ubuntu 11.10 Linux package. Structure of native and mutant Tyrp1 protein was used as starting point for MD simulations. Systems were solvated in a cubic box with simple point charge (SPC) water molecules at 10 Å marginal radius. At physiological pH the structures were found to be negatively charged; thus in order to make the simulation system electrically neutral, we added 10 sodium ions (Na+) to the simulation box using the “genion” tool that accompanies with gromacs package. Initially the solvent molecules were relaxed while all the solute atoms were harmonically restrained to their original positions with a force constant of 100 kcal/moL for 5000 steps. After this, whole molecular system was subjected to energy minimization for 5000 iterations by steepest descent algorithm implementing GROMOS96 43a1 force field. Berendsen temperature coupling method [56] was used to regulate the temperature inside the box. Electrostatic interactions were computed using the Particle Mesh Ewald method [57]. The ionization states of the residues were set appropriate to pH 7 with all histidines assumed neutral. The pressure was maintained at 1 atm with the allowed compressibility range of 4.5e − 5 atm. SHAKE algorithm was used to constrain bond lengths involving hydrogen, permitting a time step of 2 fs. Van der Waals and coulomb interactions were truncated at 1.0 nm. The nonbonded pair list was updated every 10 steps and conformations were stored every 0.5 ps. Position restraint simulation for 500 ps was implemented to allow solvent molecules to enter the cavity region of structure. Finally, systems were subjected to MD simulation for 20 ns. We then computed the comparative analysis of structural deviations in native and mutant structure. RMSD, RMSF, SASA, Rg, DSSP, and density plot analysis were carried out by using g_rms, g_rmsf, g_sas, g_gyrate, do_dssp, and g_density tool, respectively. Number of distinct hydrogen bonds formed by specific residues to other amino acids within the protein during the simulation (NH bond) was calculated using g_hbond. NH bond determined on the basis of donor-acceptor distance smaller than 0.35 nm and of donor-hydrogen-acceptor. All the graphs were plotted using XMGRACE [58] program.

### 2.5. Principal Component Analysis

The calculation of the eigenvectors and eigenvalues, and their projection along the first two principal components, was carried out using essential dynamics (ED) method according to protocol [59] within the GROMACS software package. The principle component analysis or ED is a technique that reduces the complexity of the data and extracts the concerted motion in simulations that are essentially correlated and presumably meaningful for biological function [59]. In the ED analysis, a variance/covariance matrix was constructed from the trajectories after removal of the rotational and translational movements. A set of eigenvectors and eigenvalues was identified by diagonalizing the matrix. The eigenvalues represents the amplitude of the eigenvector along the multidimensional space, and the displacement of atoms along each eigenvector shows the concerted motions of protein along each direction. The movements of structures in the essential subspace were identified by projecting the Cartesian trajectory coordinates along the most important eigenvectors from the analysis. Backbone C-alpha bonds trajectories were obtained using g_covar and g_anaeig of gromacs utilities.

## 3. Results and Discussion

To determine the deleterious nonsynonymous single nucleotide polymorphisms (nsSNPs), which might be involved in inducing disease associated phenomena, is now among the most important field of computational genomic research. The disease associated mutations can be identified with the help of genome sequencing and its analysis. The advanced method in computational biology has now enabled us to determine the deleterious nsSNPs in the target candidate genes. Computational methods were applied to study the protein structural and functional effect on point mutation at molecular level. In this investigation we implemented multiple computational methods to identify the most likely pathogenic mutations in TYRP1 gene. Our results also revealed that implementations of different algorithms often serve as powerful tools for prioritizing candidate functional nsSNPs. Here we used SIFT, PolyPhen, I-Mutant 3.0, PANTHER, PhD-SNP, SNP&GO, Pmut, and MutPred tools to examine the most deleterious and disease associated nsSNPs from the SNP dataset. The combination of methods based on evolutionary information and protein structure and/or functional parameters were used in order to increase the prediction accuracy. 

### 3.1. Screening of Deleterious nsSNPs by PolyPhen 2.0, SIFT, I-Mutant 3.0, and PANTHER Program

Out of 63 input polymorphic dataset, 42 nsSNPs were found to be “damaging” (0.5 to 1.000) to protein structure and function and the remaining 21 nsSNPs were characterized as benign by PolyPhen 2.0. Among these 42 deleterious nsSNPs, 15 SNPs G63S, W249G, C303G, Y522C, G300E, A380S, V189L, R153C, N132I, N435H, R374G, D343V, T262M, R326H, and R356Q were reported to be highly deleterious with PolyPhen score of 1.000 (Table 1). In SIFT, 34 mutations (G63S, M266T, R146W, W249G, A486T, R73W, T366M, C303G, Y522C, A380S, S305R, G309E, V189L, A409V, L7P, D123V, A31G, N132I, F383L, N96Y, N435H, R114C, G309R, R471W, D343V, G174L, A24T, T262M, R93H, R505C, V319G, R326H, R93C, and R356Q) were predicted to be deleterious with tolerance index ≥0.05 (Table 1). Among these, 17 mutations G63S, M266T, W249G, C303G, G309E, V189L, N132I, N96Y, N435H, R114C, G309R, R471W, D343V, G174L, T262M, R326H, and R356Q were reported to be highly deleterious with SIFT score of 0.00 (Table 1). Furthermore, 29 mutations were identified as deleterious and damaging in SIFT and PolyPhen 2.0 server (Table 1) which also shows a strong correlation between the prediction methodologies implemented by these two servers. SIFT and PolyPhen were shown to have better performance in identifying functional nsSNPs among other *in silico* tools [60]. The accuracy of SIFT and PolyPhen was further validated through our results, which makes these tools more suitable for the prediction [61]. All the nsSNPs submitted to PolyPhen 2.0 and SIFT were also submitted as input to the I-Mutant 3.0 server. 45 mutations were predicted to affect the stability of the protein structure by I-Mutant 3.0. Remaining out of 18 mutations, 16 mutations showed the neutral effect on protein structure and 2 mutations showed increased stability of the structure. To further validate these results we implemented HMM based statistical prediction method to identify the functionally significant point mutations using PANTHER server. The mutations with subPSEC score less than −3 have been reported to be probably deleterious. 39 mutations with subPSEC score less than or equal to −3 were characterized to be deleterious. We filtered 19 mutations (M266T, R146W, W249G, C303G, Y522C, G309E, V189L, A409V, F383L, N435H, G309R, D343V, G174L, T262M, R93H, V319G, R326H, R93C, and R356Q) which were commonly predicted to be deleterious and damaging by SIFT, PolyPhen 2.0, I-Mutant 3.0, and PANTHER servers (Table 1).

### 3.2. Prediction of Disease-Associated nsSNPs

We applied PhD-SNP which is based on support vector machine tool to further classify the predicted deleterious nsSNPs as disease associated. Total 19 nsSNPs which were commonly predicted in SIFT, PolyPhen 2.0, I-Mutant 3.0, and PANTHER tools were further used in PhD-SNP server. Out of 19 mutations, 16 of them (R146W, C303G, G309E, V189L, A409V, F383L, N435H, G309R, D343V, G174L, T262M, R93H, V319G, R326H, R93C, and R356Q) were predicted to be disease associated (Table 2). In SNP&GO, 19 nsSNPs were predicted to be disease associated. To verify this prediction, we further employed artificial neural network (ANN) based Pmut tool. Out of 19 nsSNPs, 10 mutations showed pathogenecity and remaining 9 nsSNP showed as neutral (Table 2). Particularly, R326H showed higher pathogenecity level with pathogenicity index of 0.9314 (Table 2). 8 mutations (R146W, G309E, G309R, D343V, T262M, R93H, R326H, and R356Q) were predicted as most disease associated by PhD-SNP, SNP&GO, and Pmut servers. These 8 mutations were further analysed by MutPred tool to predict the SNP disease-association probability and probable change in the molecular mechanism in the mutant. We found R326H to be highly deleterious with general probability (*g*) scores of 0.938 and was predicted to induce the loss of stability with (*p*) score of 0.0202, showing confident hypothesis. R356Q was found to be highly deleterious with general probability (*g*) scores of 0.801 and was predicted to induce the loss of catalytic residue at R356 with (*p*) score of 0.0446, showing confident hypothesis. At the end of so many mutations considered, we screened R326H and R356Q as the most deleterious and disease associated mutation in TYRP1 gene (Table 3). This prediction could be endorsed with the observed experimental data [62]. 

### 3.3. Modelling of Protein

The human Tyrp1 protein (between domain regions 190–385) was modelled by automated protein structure prediction program, I-TASSER [53]. The program used more than ten templates to model the protein. The top most template (PDB ID: 3nm8A) has covered 86% of the Tyrp1 protein query sequence. The best structure with high confidence score was collected and used for further investigations. The disease associated mutations of R326H and R356Q can probably alter the native conformation of the Tyrp1 protein structure. Hence we made a point mutation in native Tyrp1 protein at the position of 326 (arginine to histidine) and 356 (arginine to glutamine) to build the mutant structures. The quality of the modeled structure of native and mutant Tyrp1 protein was evaluated independently by the PROCHECK [54] and PROSA [55] programs, which showed good stereochemical properties of the modeled proteins. Native Tyrp1 protein showed 91.7% of residues in most favoured and allowed region and *z*-score value of −3.4. Mutant of R326H showed 92.3% of residues in most favoured and allowed region and *z*-score value of −2.13. Mutant of R356Q showed 92.3% of residues in most favoured and allowed region and *z*-score value of −2.81. Native and mutant (R326H and R356Q) Tyrp1 structures showed the *g*-factor in the ranges of 0.40, 0.45, and 0.46, respectively. The overall *G*-factors of native and mutant Tyrp1 protein structures (acceptable between 0 and 0.5) were produced by PROCHECK in the range of 0.40–0.46. These scores implicate high confidence level and hence the structures were selected for further MD analysis.

### 3.4. Molecular Dynamics Simulation

**Figure 2:**
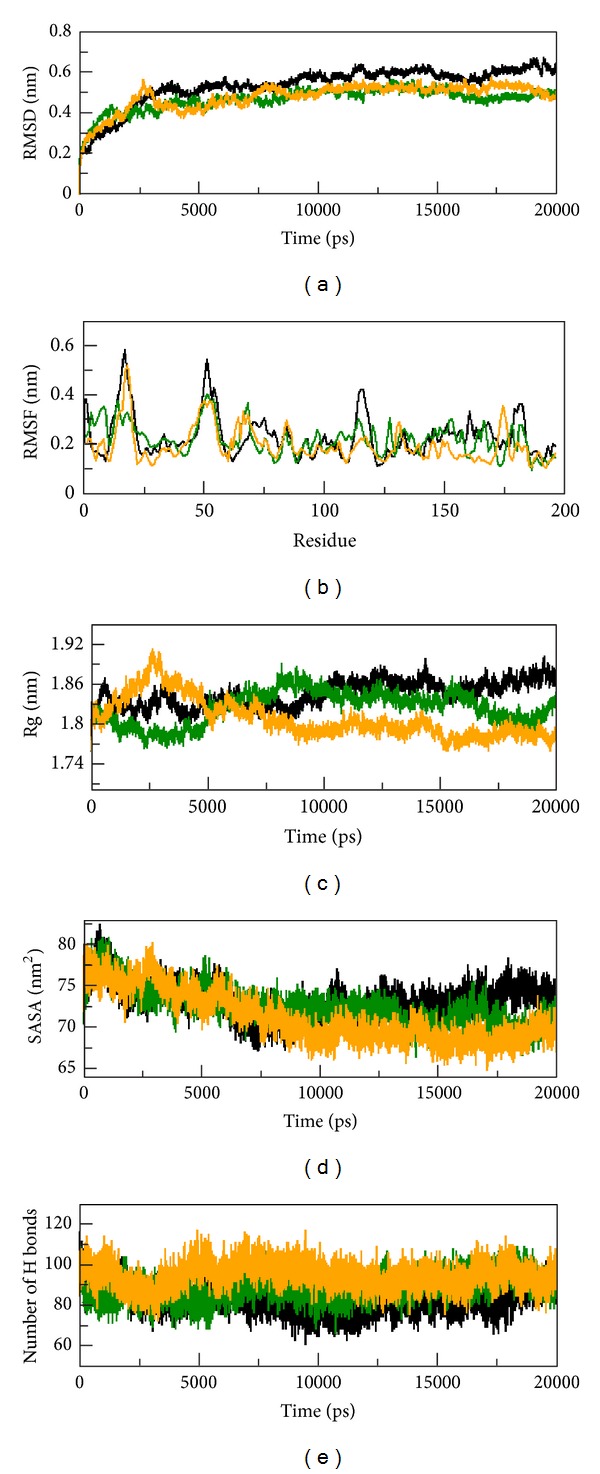
RMSD, RMSF, Rg, SASA, and NH bond of native and mutant Tyrp1 proteins versus time at 300 K. Native is shown in black, mutant (R326H) in green, and mutant (R356Q) in yellow.

To understand the structural and functional behaviour of the prioritized disease associated mutations, we performed molecular dynamics simulation for native and mutant Tyrp1 proteins. The following seven factors, namely, tolerance index, PSIC score, DDG value, subPSEC score, disease-association study, pathogenecity index, general score (*g*), and property score (*p*), which correspond to conformational changes in protein residues due to the mutation, may lead to affect the functional behaviour of Tyrp1 protein. The results obtained from above analysis further inspired us to study the dynamic behaviour of native and mutant (R326H and R356Q) Tyrp1 proteins. We studied RMSD, RMSF, Rg, SASA, and NH bond variations, DSSP, density plot, and ED analysis between the native and mutant (R326H and R356Q) Tyrp1 protein structures. Further, the RMSD for all C*α* atoms from the initial structure was examined to study the convergence of the protein system. In Figure 2(a), native and both mutant (R326H and R356Q) structures showed similar way of deviation till 3050 ps from their starting structure, resulting in a backbone RMSD of ~0.14 to 0.52 nm during the simulations. After this, native structure retained the maximum deviation till the end of the simulation resulting in the backbone RMSD of ~0.51 to ~0.66 nm, respectively. R326H and R356Q mutant structures showed the minimum deviation till the end of the simulation, resulting in the backbone RMSD of ~0.37 to ~0.51 nm and ~0.38 to ~0.54 nm, respectively. 

This magnitude of fluctuations, together with very small difference between the average RMSD values after the relaxation period (~0.52 nm), leads to produce stable trajectories in simulation, and it provided an appropriate basis for further analysis. The average value of RMSD during the simulation time period both native and mutant (R326H and R356Q) structures is signified in Table 4. Through the aim of determining RMSF we predicted whether the mutation disturbs the dynamic behaviour of residues. The RMSF values of native and mutant (R325H and R356Q) structures were collected and shown in Figure 2(b). Analysis of fluctuation score depicted that the higher degree of flexibility was observed in native structure than mutant (R326H and R356Q) Tyrp1 protein structures. The radius of gyration (Rg) is defined as the mass-weight root-mean-square distance of collection of atoms from their common center of mass. Therefore it provides an insight into the overall dimension of the protein. Radius of gyration plot for C*α* atoms of protein versus time at 300 K is shown in Figure 2(c).

In Figure 2(c), at the end of the simulation native structures showed greater Rg value than mutant (R326H and R356Q) structures. The native structure showed Rg value of ~1.76 nm at 0 ps, ~1.82 nm at 4000 ps, ~1.82 nm at 9500 ps, ~1.85 nm at 11800 ps, ~1.86 nm at 14600 ps, ~1.86 nm at 17500 ps, and ~1.87 nm at 20000 ps. R326H mutant structure showed Rg value of ~1.76 nm at 0 ps, ~1.78 nm at 4000 ps, ~1.86 nm at 9500 ps, ~1.84 nm at 11800 ps, ~1.82 nm at 14600 ps, ~1.81 nm at 17500 ps, and ~1.83 nm at 20000 ps. R356Q mutant structure showed Rg value of ~1.77 nm at 0 ps, ~1.84 nm at 4000 ps, ~1.79 nm at 9500 ps, ~1.79 nm at 11800 ps, ~1.80 nm at 14600 ps, ~1.77 nm at 17500 ps, and 1.78 nm at 20000 ps, respectively. The average Rg value was 1.84 nm in native, whereas the mutant R326H and R356Q structures showed average Rg value of 1.81 and 1.79 nm, respectively, signified in Table 4. A notable change was observed in both mutant (R326H and R356Q) structures as compared to the native. The change of SASA for native and mutant (R326H and R356Q) proteins with time is shown in Figure 2(d). Solvent accessibility surface area accounts for bimolecular surface area that is assessable to solvent molecules. Decreased value of SASA in mutant structures denotes its relatively shrunken nature as compared to the native structure. The change of SASA of native and mutant proteins with time is shown in Figure 2(d). Native and mutant (R326H and R356Q) structures showed similar fashion of deviation till 12000 ps from the initial structure, but after this native structure showed greater value of SASA than mutant (R326H and R356Q) structures. The average SASA value was 74.5 in native, whereas the mutant (R326H and R356Q) structures showed average SASA value of 71.2 and 69.1, respectively, as depicted in Table 4. We also observed notable differences in NH bond pattern during simulation, whereas the native structure showed less participation in NH bonds formation with other amino acid, while in mutant (R326H and R356Q) structures there was greater number of NH bonds (Figure 2(e)). The average value of NH bond was signified in Table 4. 

The NH bond results of native and mutant Tyrp1 structure according to the RMSD, RMSF, Rg, and SASA plot results depicted that the mutant (R326H and R356Q) structural conformation became rigid upon mutation which may lead to disturb the functional behaviour of the protein. This was further supported by the atomic density plot and PCA analysis. The consequences of these molecular changes were clearly observed in the atomic density distribution plot. There was a significant change in density distribution in mutant as compared to the native and it was depicted. Moreover the native structure shows highest atomic density distribution of 41.9 nm^−3^ but mutant (R326H and R356Q) structures showed 52.2 and 52.3 nm^−3^, respectively, (Table 4) (Figure S1a-c available online at: http://dx.doi.org/10.1155/2013/697051). It was further indicated that native structure has more flexibility than mutant (R326H and R356Q) structures.

**Figure 3:**
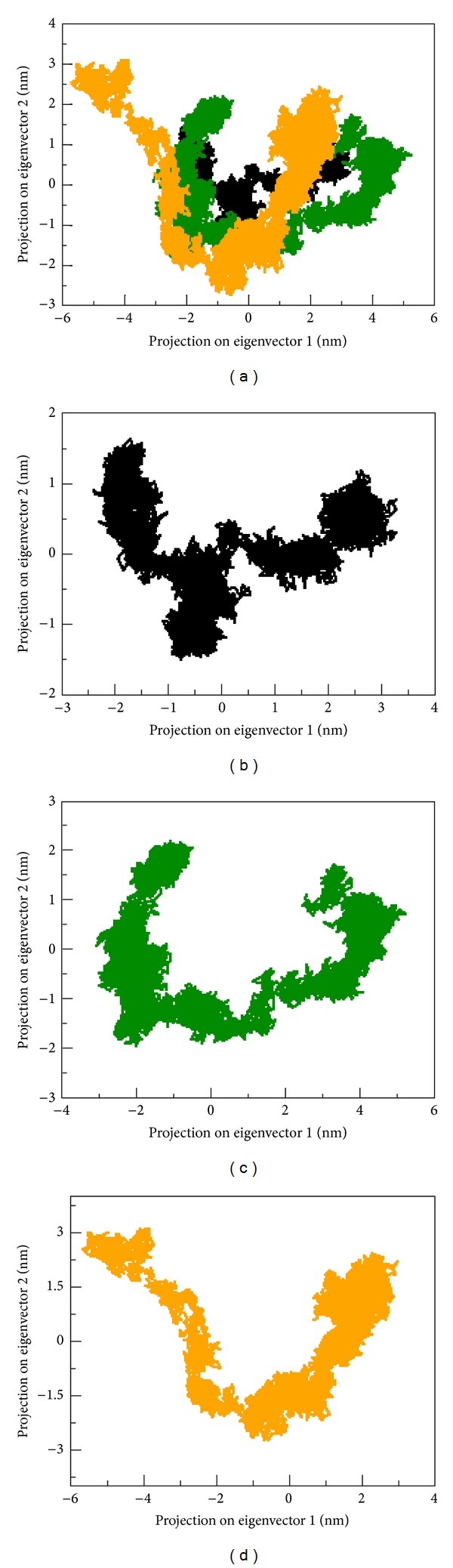
Projection of the motion of the protein in phase space along the first two principal eigenvectors at 300 K. (a) Native is shown in black, mutant (R326H) in green, and mutant (R356Q) in yellow. For clarity's sake, each trajectory is also shown separately in (b), (c), (d).

The spectrum of the corresponding eigenvalues indicated the level of fluctuation and dynamic behaviour of protein molecule in the system and was basically confined within the first two eigenvectors. Both mutant (R326H and R356Q) structures covered a small region of phase space particularly along PC1 plane than native (Figure 3(a)). Overall flexibility of two proteins was further examined by the trace of the diagonalized covariance matrix of the C*α* atomic positional fluctuations. We obtained the following values for native and mutant (R326H and R356Q) structures: 11.56 nm^2^, 10.74 nm^2^, and 8.16 nm^2^, respectively (Table 4), and again it was confirming the overall increased flexibility in native than mutant (R326H and R356Q) structures at 300 K. 

**Figure 4:**
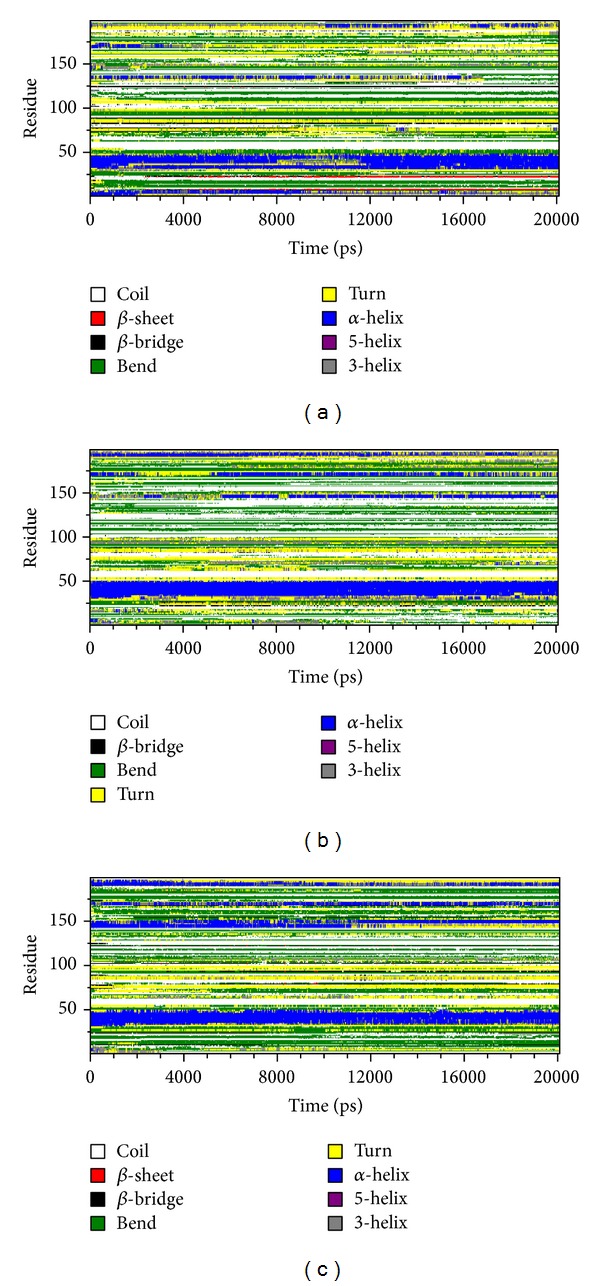
Time evolution of the secondary structural elements of the native and mutant (R326H and R356Q) Tyrp1 proteins at 300 K (DSSP classification). (a) Native, (b) mutant R326H, and (c) mutant R356Q.

We applied the DSSP algorithm [63] to monitor changes in secondary structure during the simulations. As shown in Figures 4(a)–4(c), a difference is observed only at the level of Helix from the amino acid residual position of 150 to 175 and the level of sheet from the amino acid residual position of 5 to 20. In DSSP, the most significant structural change was an increase in helical content and absence in *β*-sheet, which was observed in both mutant (R326H and R356Q) structures. 

**Figure 5:**
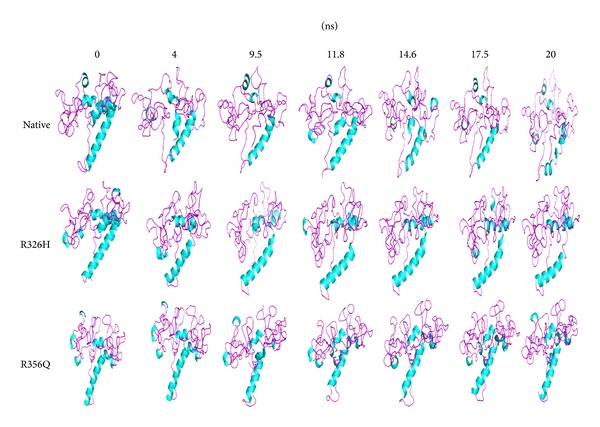
Snapshots of native and mutant (R326H and R356Q) Tyrp1 protein conformation at different simulation time steps.

To examine how the structure got damaged and leads to affect the functions upon mutation, we analysed the native and mutant (R326H and R356Q) structures at different time scales (Figure 5). It was clearly observed that there is continuous loss of helix in native structure than mutant structures till the end of the simulation. This was well supported by the DSSP analysis. It indicates that both mutant (R326H and R356Q) structures showed an increase in helical content and total absence in *β*-sheet in the amino acid residual position from 150 to 170 and 5 to 20, respectively. In general, helices are mostly rigid, whereas spanning loop regions are mostly flexible. [64–66]. Based on that here, both mutant structures (R326H and R356Q) showed more helical content which leads to more rigidity in the conformation. On the basis of DSSP analysis, it was confirmed that due to mutation the Tyrp1 structure became rigid. Therefore, it seems evident that both mutations (R326H and R356Q) have cruel damaging impact on protein structural orientation and its function. This prediction is also endorsed with the observed experimental data [62, 67]. This study provides an essential insight into the underlying molecular mechanism of Tyrp1 protein upon mutation and in future it may help to develop a personalized medicine for OCA3.

## 4. Conclusion

Computational analysis has now become a roadmap to define a standard disease specific SNP at molecular level. In this study we screened two most disease associated mutations (R326H and R356Q) which are related to OCA3. Molecular dynamics simulation approaches have also been extensively used to report the structural consequences of the deleterious predicted point mutations. The flexibility loss is observed in RMSD, RMSF, Rg plot which is further supported by a decrease in SASA value in both mutant structures. This may produce a major impact on the structural conformation of Tyrp1 protein, which also affects the function of Tyrp1 protein. Due to mutation, the structure became more rigid which is also supported by NH bond, density plot, PCA, and DSSP analysis. Our result suggests a significant computational roadmap to detect the OCA3 associated SNPs from the large SNP dataset and reduce the expenses in experimental depiction of pathological nsSNPs. Further the predicted R326H and R356Q mutations can be further studied by wet lab scientist to investigate the evidence of Tyrp1 protein mutation in association to OCA3 and develop a potent drug target for OCA3.

## Supplementary Material

The consequences upon mutations were clearly observed in the atomic density distribution plot. There was a significant change in density distribution in mutant as compared to the native. Native structure shows highest atomic density distribution of 41.9 nm^−3^ but in mutant (R326H & R356Q) structures showed 52.2 and 52.3 nm^−3^ respectively. It was further indicate that native has more flexibility than mutant (R326H & R356Q) structures.

## Figures and Tables

**Table 1:** nsSNPs analyzed by four computational methods PolyPhen 2.0, SIFT, I-Mutant 3.0, and PANTHER in TYRP1 gene.

SNP ID	Mutation	PolyPhen 2.0		SIFT		I-MUTANT 3.0		PANTHER	
		PSIC	Prediction	Score	Prediction	DDG	Stability	subPSEC	Prediction
rs202189890	S270R	0.996	Damaging	0.18	Tolerated	−0.43	Neutral	−2.23829	Tolerated
rs202126779	T253M	0.996	Damaging	0.08	Tolerated	−0.32	Decrease	−3.02723	Deleterious
rs201899938	S8F	0.963	Damaging	0.7	Tolerated	0.20	Neutral	−2.19231	Tolerated
rs201789348	E139K	0.301	Benign	0.24	Tolerated	−0.97	Decrease	−3.62832	Deleterious
rs201605146	S470N	0.134	Benign	0.36	Tolerated	−0.48	Neutral	−2.11901	Tolerated
rs201457510	G63S	1.000	Damaging	0.00	Deleterious	−1.17	Neutral	−4.06048	Deleterious
rs201345670	**M266T**	0.772	Damaging	0.00	Deleterious	−1.63	Decrease	−3.93879	Deleterious
rs201293896	**R146W**	0.975	Damaging	0.02	Deleterious	−0.36	Decrease	−5.75453	Deleterious
rs200882524	L487F	0.998	Damaging	0.23	Tolerated	−0.98	Decrease	−4.01429	Deleterious
rs200754545	**W249G**	1.000	Damaging	0.00	Deleterious	−2.43	Decrease	−7.90178	Deleterious
rs200607153	A486T	0.029	Benign	0.05	Deleterious	−0.56	Decrease	−2.56844	Tolerated
rs199989943	R73W	0.999	Damaging	0.02	Deleterious	−0.24	Neutral	−5.69984	Deleterious
rs199823942	T366M	0.014	Benign	0.01	Deleterious	−0.43	Decrease	−4.14092	Tolerated
rs193035382	**C303G**	1.000	Damaging	0.00	Deleterious	−1.49	Decrease	−3.02248	Deleterious
rs188236569	**Y522C**	1.000	Damaging	0.01	Deleterious	−0.79	Decrease	−4.39247	Deleterious
rs187959351	G300E	1.000	Damaging	0.86	Tolerated	−0.39	Decrease	−3.66042	Deleterious
rs184910238	A380S	1.000	Damaging	0.05	Deleterious	−0.66	Decrease	−2.88075	Tolerated
rs183546444	P476T	0.000	Benign	1.00	Tolerated	−1.13	Decrease	−2.05148	Tolerated
rs182508840	T275A	0.064	Benign	0.10	Tolerated	−1.58	Decrease	−2.92255	Tolerated
rs181755026	E524A	0.001	Benign	0.22	Tolerated	−0.45	Decrease	−2.15096	Tolerated
rs151091299	S305R	0.992	Damaging	0.01	Deleterious	−0.22	Neutral	−3.02846	Deleterious
rs150899857	R505H	0.753	Damaging	0.07	Tolerated	−1.32	Decrease	−3.32609	Deleterious
rs149856598	**G309E**	0.566	Damaging	0.00	Deleterious	−0.80	Decrease	−3.82367	Deleterious
rs149324507	**V189L**	1.000	Damaging	0.00	Deleterious	−0.79	Decrease	−3.93115	Deleterious
rs148777155	S498P	0.761	Damaging	0.19	Tolerated	−0.27	Neutral	−4.76166	Deleterious
rs148248971	**A409V**	0.972	Damaging	0.01	Deleterious	−0.02	Decrease	−3.75227	Deleterious
rs147212712	L7P	0.924	Damaging	0.01	Deleterious	−1.44	Decrease	NA	NA
rs146962444	D123V	0.628	Damaging	0.03	Deleterious	−0.34	Neutral	−3.42156	Deleterious
rs146838872	V33A	0.000	Benign	0.45	Tolerated	−0.86	Decrease	−2.4943	Tolerated
rs146467307	V373I	0.013	Benign	0.54	Tolerated	−0.61	Decrease	−2.69258	Tolerated
rs146027807	R153C	1.000	Damaging	0.05	Tolerated	−0.90	Decrease	−5.95016	Deleterious
rs144438412	A67T	0.001	Benign	0.36	Tolerated	−0.69	Decrease	−2.56649	Tolerated
rs144206983	S207I	0.968	Damaging	0.24	Tolerated	0.43	Increase	NA	NA
rs143705229	A31G	0.159	Benign	0.02	Deleterious	−1.39	Decrease	−4.24808	Deleterious
rs143610120	N132I	1.000	Damaging	0.00	Deleterious	0.82	Increase	−6.81596	Deleterious
rs143106698	**F383L**	0.999	Damaging	0.04	Deleterious	−0.99	Decrease	−3.52387	Deleterious
rs143004036	N96Y	0.987	Damaging	0.00	Deleterious	−0.20	Neutral	−4.96986	Deleterious
rs142019860	Q520K	0.067	Benign	0.05	Tolerated	−0.16	Decrease	−2.41741	Tolerated
rs141949653	**N435H**	1.000	Damaging	0.00	Deleterious	−0.72	Decrease	−4.94266	Deleterious
rs141834891	R23W	0.02	Benign	0.22	Tolerated	0.13	Neutral	−2.04234	Tolerated
rs141196295	R114C	0.019	Benign	0.00	Deleterious	−0.68	Neutral	−4.80738	Deleterious
rs140233627	S506G	0.004	Benign	0.40	Tolerated	−0.96	Decrease	−2.00879	Tolerated
rs139946740	**G309R**	0.754	Damaging	0.00	Deleterious	−0.56	Decrease	−5.11275	Deleterious
rs139670838	R55H	0.771	Damaging	0.13	Tolerated	−1.12	Decrease	−3.93481	Deleterious
rs138272660	R471W	0.986	Damaging	0.00	Deleterious	−0.23	Neutral	−5.61942	Deleterious
rs138038972	R125K	0.000	Benign	0.21	Tolerated	−1.00	Neutral	−2.33412	Tolerated
rs121912778	R374G	1.000	Damaging	0.06	Tolerated	−1.48	Decrease	−3.98421	Deleterious
rs113146199	**D343V**	1.000	Damaging	0.00	Deleterious	0.14	Decrease	−4.03201	Deleterious
rs78071458	**G174L**	0.993	Damaging	0.00	Deleterious	−0.29	Decrease	−4.71688	Deleterious
rs61758405	A24T	0.219	Benign	0.02	Deleterious	−0.52	Decrease	−3.03529	Deleterious
rs61752939	**T262M**	1.000	Damaging	0.00	Deleterious	−0.34	Decrease	−5.00198	Deleterious
rs61752937	**R93H**	0.998	Damaging	0.04	Deleterious	−1.60	Decrease	−3.09393	Deleterious
rs61752864	A70T	0.859	Damaging	0.09	Tolerated	−0.66	Decrease	−2.58273	Tolerated
rs41306053	D308N	0.000	Benign	0.70	Tolerated	−1.12	Decrease	−2.04757	Tolerated
rs41305647	Q518H	0.000	Benign	0.23	Tolerated	−0.43	Decrease	−2.67099	Tolerated
rs41305645	Q530R	0.029	Benign	0.43	Tolerated	−0.07	Neutral	−2.1177	Tolerated
rs41303653	G485A	0.000	Benign	1.00	Tolerated	−0.63	Neutral	−3.35217	Deleterious
rs41303651	R505C	0.861	Damaging	0.01	Deleterious	−0.89	Neutral	−2.64052	Tolerated
rs35197549	**V319G**	0.997	Damaging	0.01	Deleterious	−1.86	Decrease	−3.20938	Deleterious
rs16929374	**R326H**	1.000	Damaging	0.00	Deleterious	−1.21	Decrease	−4.86382	Deleterious
rs3202399	E413K	0.976	Damaging	0.18	Tolerated	−1.07	Decrease	−2.76125	Tolerated
VAR_068176	**R93C**	0.999	Damaging	0.01	Deleterious	−1.24	Decrease	−4.10005	Deleterious
VAR_026828	**R356Q**	1.000	Damaging	0.00	Deleterious	−1.49	Decrease	−5.86235	Deleterious

SNPs highlighted in bold are predicted to be deleterious.

**Table 2:** The disease associated SNPs are predicted from PHDsnp, SNP&GO, and Pmut servers.

SNP IDs	Mutation	PHDsnp results	SNP&GO	Pmut	
				Score	Prediction
rs201345670	M266T	Neutral	Disease	0.4759	Neutral
rs201293896	**R146W**	Disease	Disease	0.6728	Pathological
rs200754545	W249G	Neutral	Disease	0.5638	Pathological
rs193035382	C303G	Disease	Disease	0.2516	Neutral
rs188236569	Y522C	Neutral	Disease	0.6752	Pathological
rs149856598	**G309E**	Disease	Disease	0.8774	Pathological
rs149324507	V189L	Disease	Disease	0.4793	Neutral
rs148248971	A409V	Disease	Disease	0.4533	Neutral
rs143106698	F383L	Disease	Disease	0.3564	Neutral
rs141949653	N435H	Disease	Disease	0.1202	Neutral
rs139946740	**G309R**	Disease	Disease	0.6503	Pathological
rs113146199	**D343V**	Disease	Disease	0.5798	Pathological
rs78071458	G174L	Disease	Disease	0.3075	Neutral
rs61752939	**T262M**	Disease	Disease	0.6624	Pathological
rs61752937	**R93H**	Disease	Disease	0.6320	Pathological
rs35197549	V319G	Disease	Disease	0.2561	Neutral
rs16929374	**R326H**	Disease	Disease	0.9314	Pathological
VAR_068176	R93C	Disease	Disease	0.1465	Neutral
VAR_026828	**R356Q**	Disease	Disease	0.6210	Pathological

Disease associated SNPs are displayed in bold.

**Table 3:** The *G* score, *P* score, molecular variations, and prediction reliability calculated from MutPred server. Here the most disease associated mutations are displayed in bold.

SNP ID	Mutation	MUTPred			
		*G* score	*P* score	Molecular Variation	Prediction reliability
rs201293896	R146W	0.524	0.0566	Loss of disorder	No reliable Inference
rs149856598	G309E	0.563	0.0869	Loss of catalytic residue	No reliable inference
rs139946740	G309R	0.611	0.0971	Gain of solvent accessibility	No reliable inference
rs113146199	D343V	0.613	0.0676	Loss of disorder	No reliable inference
rs61752939	T262M	0.599	0.079	Loss of helix	No reliable inference
rs61752937	R93H	0.354	0.0986	Loss of disorder	No reliable inference
rs16929374	**R326H**	0.938	0.0202	Loss of stability	**Confident hypothesis**
VAR_026828	**R356Q**	0.801	0.0446	Loss of catalytic residue at R356	**Confident hypothesis**

**Table 4:** Average values of RMSD, Rg, SASA, NH-bonds, trace of co-variance, and density value of native and mutant (R326H and R356Q) structures.

	Native	Mutant (R326H)	Mutant (R356Q)
RMSD	0.62	0.50	0.52
Rg	1.84	1.81	1.79
SASA	74.5	71.2	69.1
NH-bonds	84.7	93.2	92.4
Density value	41.9	52.2	52.3
Trace of Co-variance	11.56	10.74	8.16

RMSD: root-mean-square deviation; Rg: radius of gyration; SASA: solvent accessible surface area; NH bonds: number of hydrogen bonds. The value of RMSD, Rg and SASA, co-variance, and total helicity are given in nm, whereas density value is given in nm^−3^.

## Abbreviations

nsSNPs: Non-synonymous single nucleotide polymorphism
OCA3: Oculocutaneous albinism type3
Tyrp1: Tyrosinase-related protein-1
MDS: Molecular dynamics simulation
RMSD: Root-mean-square deviation
RMSF: Root-mean-square fluctuation
Rg: Radius of gyration
SASA: Solvent-accessible surface area
NH bonds: Number of hydrogen bonds
PCA: Principal component analysis
DSSP: Definition of secondary structure of proteins.
