# Supplementary figures and images for: In Silico Screening and Molecular Dynamics Simulation of Disease-Associated nsSNP in TYRP1 Gene and Its Structural Consequences in OCA3
Source: Biomed Res Int. 2013 Jun 19;2013:697051. doi: 10.1155/2013/697051 (PMC3703794; doi:10.1155/2013/697051)

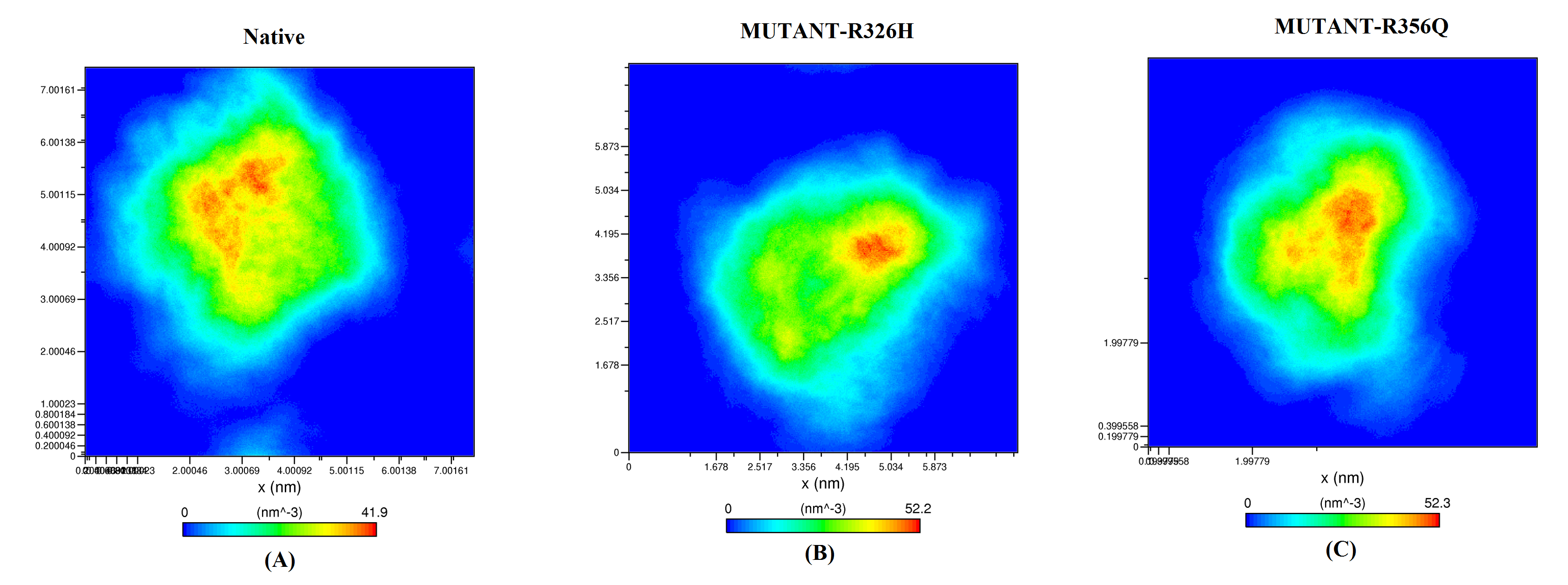

Supplement: Supplementary file 1 — The consequences upon mutations were clearly observed in the atomic density distribution plot. There was a significant change in density distribution in mutant as compared to the native. Native structure shows highest atomic density distribution of 41.9 nm−3 but in mutant (R326H & R356Q) structures showed 52.2 and 52.3 nm−3 respectively. It was further indicate that native has more flexibility than mutant (R326H & R356Q) structures. [file 697051.f1.jpg]
